# White matter hyperintensities and the mediating role of cerebral amyloid angiopathy in dominantly-inherited Alzheimer’s disease

**DOI:** 10.1371/journal.pone.0195838

**Published:** 2018-05-09

**Authors:** Seonjoo Lee, Molly E. Zimmerman, Atul Narkhede, Sara E. Nasrabady, Giuseppe Tosto, Irene B. Meier, Tammie L. S. Benzinger, Daniel S. Marcus, Anne M. Fagan, Nick C. Fox, Nigel J. Cairns, David M. Holtzman, Virginia Buckles, Bernardino Ghetti, Eric McDade, Ralph N. Martins, Andrew J. Saykin, Colin L. Masters, John M. Ringman, Stefan Fӧrster, Peter R. Schofield, Reisa A. Sperling, Keith A. Johnson, Jasmeer P. Chhatwal, Stephen Salloway, Stephen Correia, Clifford R. Jack, Michael Weiner, Randall J. Bateman, John C. Morris, Richard Mayeux, Adam M. Brickman, Dominantly Inherited Alzheimer Network

**Affiliations:** 1 Research Foundation for Mental Hygiene, Inc., New York, NY, United States of America; 2 Department of Biostatistics, Mailman School of Public Health, Columbia University, New York, NY, United States of America; 3 Psychology Department, Fordham University, Bronx, NY, United States of America; 4 Department of Neurology, Albert Einstein College of Medicine, Bronx, NY, United States of America; 5 Taub Institute for Research on Alzheimer’s Disease and the Aging Brain, College of Physicians and Surgeons, Columbia University, New York, NY, United States of America; 6 Department of Neurology, College of Physicians and Surgeons, Columbia University, New York, NY, United States of America; 7 Department of Radiology, Washington University School of Medicine, Saint Louis, MO, United States of America; 8 Department of Neurology, Washington University School of Medicine, Saint Louis, MO, United States of America; 9 Dementia Research Centre, Department of Neurodegenerative Disease, UCL Institute of Neurology, London, United Kingdom; 10 Department of Pathology and Immunology, Washington University School of Medicine, Saint Louis, MO, United States of America; 11 Department of Pathology and Laboratory Medicine, Indiana University School of Medicine, Indianapolis, IN, United States of America; 12 Centre of Excellence of Alzheimer’s Disease Research and Care, School of Exercise, Biomedical and Health Sciences, Edith Cowan University, Perth, Australia; 13 Indiana Alzheimer Disease Center and Center for Neuroimaging, Department of Radiology and Imaging Science, Indiana University School of Medicine, Indianapolis, IN, United States of America; 14 The Florey Institute, University of Melbourne, Parkville, Australia; 15 Memory and Aging Center, Keck School of Medicine of University of Southern California, Los Angeles, CA, United States of America; 16 German Center for Neurodegenerative Diseases (DZNE) München and Tübingen and Department of Nuclear Medicine, Technische Universität München (TUM), Munich, Germany; 17 Neuroscience Research Australia and University of New South Wales, Sydney, Australia; 18 Departments of Radiology and Neurology, Massachusetts General Hospital, Harvard Medical School, Boston, MA, United States of America; 19 Butler Hospital and Department of Neurology, Alpert Medical School, Brown University, Providence, RI, United States of America; 20 Department of Psychiatry & Human Behavior, Alpert Medical School, Brown University, Providence, RI United States of America; 21 Department of Radiology, Mayo Clinic, Rochester, MN, United States of America; 22 Department of Radiology and Biomedical Imaging, Center for Imaging of Neurodegenerative Diseases, San Francisco Veterans Affairs Medical Center and Departments of Psychiatry, Radiology, Medicine, and Neurology, University of California at San Francisco, San Francisco, CA, United States of America; 23 Gertrude H. Sergievsky Center, College of Physicians and Surgeons, Columbia University, New York, NY, United States of America; Nathan S Kline Institute, UNITED STATES

## Abstract

**Introduction:**

White matter hyperintensity (WMH) volume on MRI is increased among presymptomatic individuals with autosomal dominant mutations for Alzheimer’s disease (AD). One potential explanation is that WMH, conventionally considered a marker of cerebrovascular disease, are a reflection of cerebral amyloid angiopathy (CAA) and that increased WMH in this population is a manifestation of this vascular form of primary AD pathology. We examined whether the presence of cerebral microbleeds, a marker of CAA, mediates the relationship between WMH and estimated symptom onset in individuals with and without autosomal dominant mutations for AD.

**Participants and methods:**

Participants (n = 175, mean age = 41.1 years) included 112 with an AD mutation and 63 first-degree non-carrier controls. We calculated the estimated years from expected symptom onset (EYO) and analyzed baseline MRI data for WMH volume and presence of cerebral microbleeds. Mixed effects regression and tests of mediation were used to examine microbleed and WMH differences between carriers and non-carriers and to test the whether the association between WMH and mutation status is dependent on the presence of microbleeds.

**Results:**

Mutation carriers were more likely to have microbleeds than non-carriers (p<0.05) and individuals with microbleeds had higher WMH volume than those without (p<0.05). Total WMH volume was increased in mutation carriers compared with non-carriers, up to 20 years prior to EYO, after controlling for microbleed status, as we demonstrated previously. Formal testing of mediation demonstrated that 21% of the association between mutation status and WMH was mediated by presence of microbleeds (p = 0.03) but a significant direct effect of WMH remained (p = 0.02) after controlling for presence of microbleeds.

**Discussion:**

Although there is some co-dependency between WMH and microbleeds, the observed increases in WMH among mutation carriers does not appear to be fully mediated by this marker of CAA. The findings highlight the possibility that WMH represent a core feature of AD independent of vascular forms of beta amyloid.

## Introduction

There is an emerging interest in the role of small vessel cerebrovascular changes in late onset Alzheimer’s disease (AD). While there is general agreement that small vessel cerebrovascular disease contributes independently to the clinical presentation of AD by lowering clinical diagnostic thresholds or conferring additive cognitive impairment, there is considerable controversy about whether it plays a primary role in AD pathogenesis, representing a core feature of the disease. The study of the contribution of small vessel cerebrovascular disease to late onset AD is difficult because the ordering and timing of the onset of biological changes is obscured by a potentially decades-long preclinical period[1] and confounded by its strong association with age and vascular risk factors, such as hypertension. We addressed these issues previously by turning to the Dominantly Inherited Alzheimer’s Network (DIAN) study[1, 2], which enrolls individuals at 50% risk for developing AD, typically early onset, by virtue of a having a first-degree relative with an autosomal dominant, fully-penetrant genetic mutation for AD. We quantified small vessel cerebrovascular disease as the regional volume of white matter hyperintensities (WMH), areas of increased signal on T2-weighted magnetic resonance imaging (MRI), that may reflect ischemic injury[3] or other mechanisms. Increased WMH volume, particularly when distributed in posterior regions, is associated with increased risk for late onset AD and with the severity and course of the disease[4]. In DIAN, we modeled WMH severity and distribution in mutation carriers and non-carriers as a function of the estimated years to symptom onset (EYO), defined as the difference between the age of the affected first-degree relative’s age of symptom onset and the participant’s age[2]. We found that mutation carriers had reliably increased WMH volume, particularly in posterior regions, which appeared elevated as much as 22 years prior to symptom onset. We concluded that WMH burden is a distinct core feature of AD that should be considered explicitly in models of disease pathogenesis.

One outstanding issue regarding these findings relates to the presence of cerebral amyloid angiopathy (CAA), the vascular deposition of amyloid pathology. Cerebral amyloid angiopathy can cause small hemorrhagic lesions, or microbleeds, that manifest as punctate hypointense voids on gradient echo (GRE) sequences[5]. Previous work showed a relationship of WMH volume, particularly in posterior areas, with cerebral microbleeds[6] and that individuals with familiar forms of AD have comparable frequencies of microbleeds to those with late onset disease[7, 8]. The question of the extent to which CAA mediates the observed relationship between WMH and AD is critical: if WMH in the context of AD is driven by CAA, then prevention or intervention strategies could focus on vascular forms of amyloid pathology. However, if the association of WMH with AD is independent of CAA, then other mechanisms or forms of vascular pathology (e.g., ischemia, blood brain barrier damage, inflammation-mediated, etc.) become highly relevant in the conceptualization of disease pathogenesis and strategies for intervention or prevention. The purpose of this study was to examine the role of CAA, operationally-defined as the presence of cerebral microbleeds on GRE MRI, in the context of autosomal dominant AD and in the relationship we observed previously between WMH and autosomal dominant mutation status.

## Materials and methods

### DIAN study

The Dominantly Inherited Alzheimer’s Network (www.dian-info.org; NIA-U19-AG032438) is a multisite effort that includes centers in the United States, UK, Germany, Australia, Japan, Korea, and Argentina. DIAN participants are from families with a known autosomal-dominant mutation for AD, including APP, PSEN1, and PSEN2, irrespective of their own mutation status. As part of the DIAN, participants receive a baseline assessment with sampling of blood and CSF, clinical assessment, neuropsychological evaluation, and neuroimaging and are followed longitudinally with identical assessments. Full procedures for the study are described elsewhere[1, 9]. All assessment and imaging procedures were approved by the Washington University Human Research Protection Office. All participants gave written informed consent according to the Declaration of Helsinki. Local institutional review boards/ethics committees for sites that contributed data to this study include, Columbia University Medical Center Institutional Review Board; University of Pittsburgh Human Research Protection Office; UCLA Institutional Review Boards; Indiana University Institutional Review Boards; Partners Human Research Committee; Butler Hospital Institutional Review Board for Human Subjects Research; The University of South Wales Human Research Ethics Committee; University of Melbourne Office for Research Ethics and Integrity; Edith Cowan University Human Research Ethics Committee; Technical University Munich Institutional Review Board; and University College London Research Ethics Committee.

### Clinical assessment

Clinical assessments were performed by individuals who were unaware of each participant’s mutation status. The evaluation included neuropsychological testing, medical examination, administration of the Clinical Dementia Rating (CDR) scale[10], and determination of the parental age of onset. A semi-structured interview, which determined the age at which the affected parent started exhibiting symptoms, was used to determine parental age at onset[1, 2]. The difference between the participant’s age and the parental age at onset was used to establish the EYO. The EYO variable was calculated for all participants regardless of their mutation status. Participants with negative EYO are younger than their parental age at onset. Data included here were a subset from Data Freeze 6.

### MRI image processing

**Fig 1:**
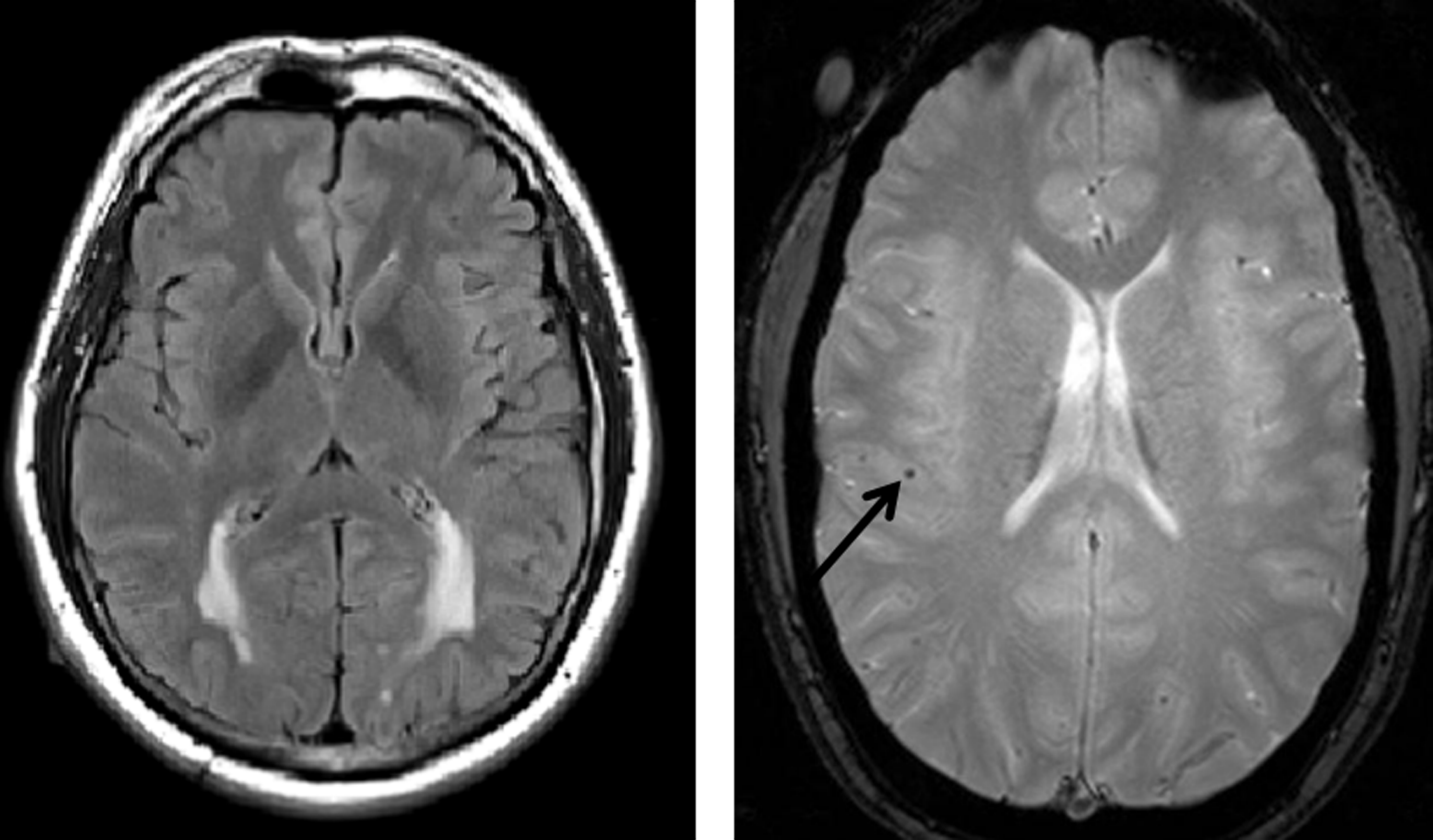
Examples of WMH and microbleeds. Left: T2-weighted FLAIR images from a mutation carrier with prominent posterior WMH, approximately 5 years prior to symptom onset. Right: example of microbleed (arrow) on a gradient echo MRI scan.

Baseline MRI data were analyzed for WMH volume on T2-weighted FLAIR scans (repetition time: 9,000; echo time: 90; voxel dimensions: 0.86 x 0.86 x 5.0 mm) and for presence and number of cerebral microbleeds, a radiological marker of CAA, on T2*-weighted scans (repetition time: 650; echo time: 20; voxel dimensions: 0.78 x 0.78 x 5.0 mm). White matter hyperintensity quantification in this cohort has been described previously[2]. Briefly, an algorithm that labeled voxel intensity values above a study-specific threshold was applied to each individual FLAIR image; the number of voxels labeled with this approach were summed and multiplied by voxel dimensions to yield total WMH volumes for each participants. We also co-registered a “lobar” atlas to each image to derive the WMH volume in the frontal, temporal, parietal, and occipital lobes. Because the values were not normally distributed, total and regional WMH volumes were transformed with inverse hyperbolic sine. Fig 1 displays an example of a participant with prominent posterior WMH.

Microbleeds, sometimes referred to as “microhemorrhages,” were identified on GRE images following procedures that have been described previously (85% interrater agreement, κ = 68%) [11]. Briefly, expert operators visually inspected each image and rated microbleeds as homogenous hypointense lesions up to 10 mm in diameter in grey or white matter. We dichotomized the participants into those with (present) and without (absent) one or more cerebral microbleeds for statistical analyses. Fig 1 displays an example of a cerebral microbleed in a DIAN participant.

### Statistical analysis

SAS (version 9.4) was used for all statistical analyses. Demographic and clinical variables were compared by microbleed status with Wilcoxson’s test and chi-squared analysis for continuous and categorical variables, respectively. We also tested whether the difference between those with and without microbleeds persisted after controlling for EYO and CDR-Sum-of-boxes (CDR-SB) using linear regression and logistic regressions for continuous and categorical variables. We used linear mixed-effect regression analysis to test whether WMH volume differed by mutation type and microbleed status, controlling for participant family as a random effect.

We also tested whether there is inflection point in microbleed status by the EYO using piece-wise generalized linear mixed-effect regression. The final models were selected using Bayesian information criteria as described elsewhere[2]. The same inflection point analyses were carried out for WMH volume with and without microbleed status as a covariate.

**Fig 2:**
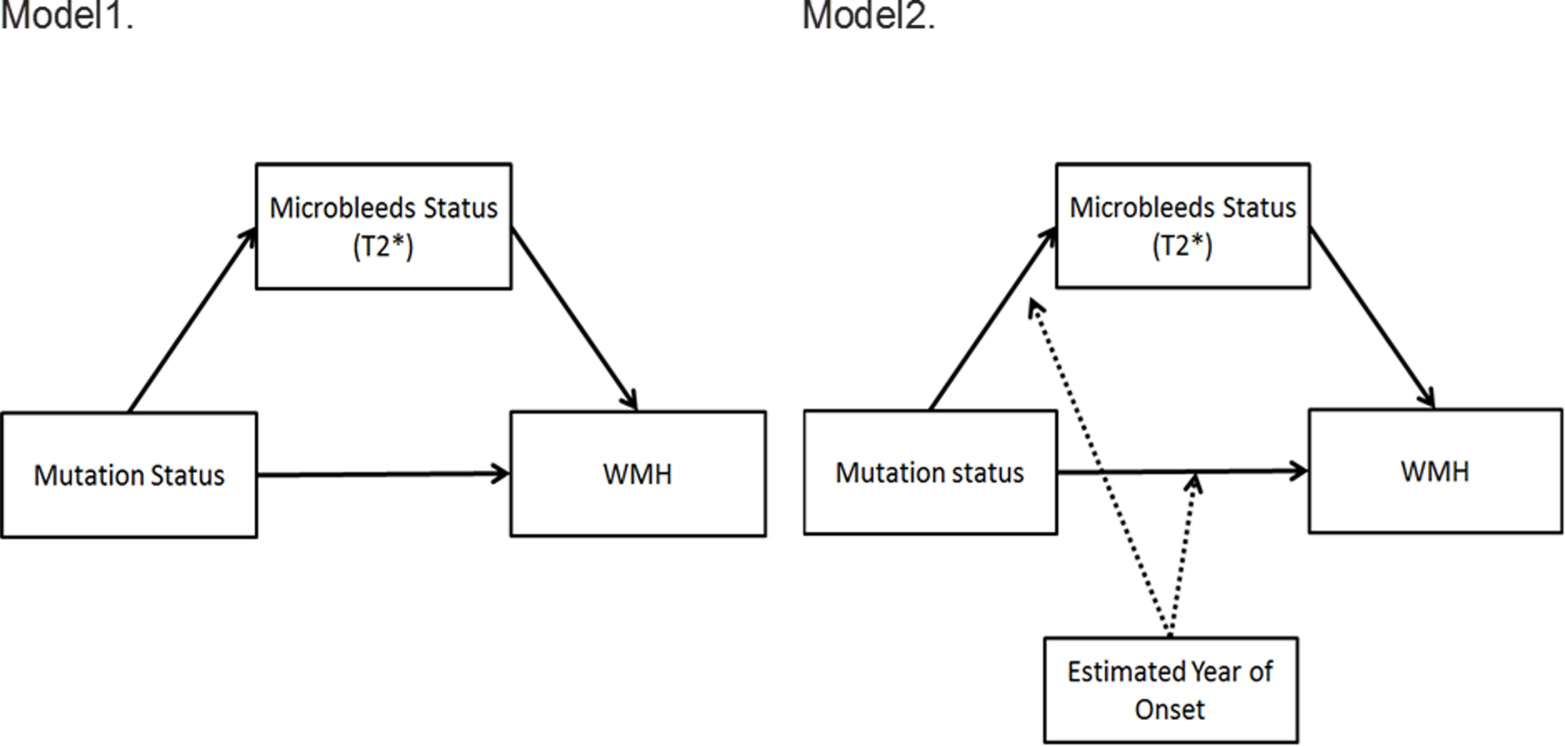
Mediation models.

Two models described in Fig 2 were tested for the primary hypotheses. Model 1 tested whether microbleed status is in the causal pathway between mutation status and WMH volume using mediation analysis. Model 2 tested whether the relationships between mutation status, microbleed status, and WMH volume differ by EYO (moderation). We separately tested whether three paths (between mutation status and WMH, between mutation status and microbleed status, and between microbleed status and WMH) differ by EYO. We did not find significant moderation effect of EYO on the association between microbleed status and WMH volume. Thus, in the final model, the moderation effect on the association between microbleed status and WMH volume was omitted. Based on causal effects models[12–14], the mediation (or indirect) effect explained by microbleed status was estimated as the difference between the total effect of mutation status on WMH and direct effect of mutation status on WMH volume controlling for microbleed status. For statistical inference, bootstrapping was used to derive 95% confidence intervals of the total, direct, and indirect effects. All models were adjusted for APOE-ε4 status, EYO and age. Models were also re-run with CDR-SB as a covariate and in the subset of participants with CDR-SB scores of less than or equal to 0.5 (n = 138) and after excluding individuals with APP mutations (n = 37) because of known associations between APP and CAA[15].

As a secondary exploratory analysis, we tested the same mediation and moderated-mediation of the number of microbleeds on the relationship between mutation status and WMH volume in the participants with one or more microbleeds (n = 26). The number of microbleeds was log-transformed to reduce skewness.

## Results

### Participants

One hundred seventy-five participants had both WMH measures and GRE MRI scans with derived data of microbleed status. There were 112 mutation carriers of whom 34 (30%) had CDR-SB scores greater than 1, indicating some degree of manifest symptomatology. There was a positive association between CDR-SB and EYO (spearman’s correlation ρ = 0.55, p<0.0001) within the 112 mutation carriers, indicating that CDR-SB scores increased with closer proximity to the estimated time of symptom onset.

### Microbleeds

Data comparing those with and without microbleeds are presented in Table 1. Among the 175 participants, 26 participants (15%) had at least one microbleed. Thirteen of 26 participants (50%) had one microbleed, 12 participants had 2–29 microbleeds and one participant had 135 microbleeds. Individuals with microbleeds were 4.6 years older, 4.5 years closer to the estimated age of onset, and had 1.61 points higher CDR-SB scores than the 146 participants without microbleeds. These 26 participants had a more severe biomarker profile than those without, including increased total WMH and WMH volume in temporal, parietal, and occipital lobes, and decreased CSF Aβ1–42, and CSF Aβ1-42/tau ratio values. These differences persisted after controlling for EYO and CDR-SB, except for the difference in CSF Aβ1-42/tau ratio values. Mutation carriers were more likely to have microbleeds than non-carriers (19.6% vs. 6.3%, Fisher’s exact test p = 0.025). This association remained significant (OR = 4.87, 95% CI: 1.16,20.51; p = 0.0334) after adjustment for age, EYO and APOE-ε4 status. Age and APOE- ε4 status were not associated with microbleed status (all p’s >0.15).

**Table 1:** Demographic, clinical, and biomarker data in microbleed presence and absence.

		All (n = 175)		Microbleed Absent (n = 149)		Microbleed Present (n = 26)		p-value
Mutation Carriers, n (%)^*^		112	(64.00)	90	(60.4)	22	(84.62)	0.0252
Age, mean years±SD^*^		41.05	±9.66	40.36	±9.43	45.00	±10.19	0.0234
EYO, mean years±SD^*^		-5.14	±9.32	-5.81	±9.29	-1.31	±8.70	0.0227
Women, n (%)		100	(57.14)	87	(58.39)	13	(50)	0.5204
Vascular factors (%)	Hypertension	20	(11.43)	15	(10.07)	5	(19.23)	0.1854
	Hypercholesterolemia	27	(15.61)	21	(14.29)	6	(23.08)	0.2508
	Diabetes	2	(1.14)	1	(0.67)	1	(3.85)	0.2758
	Smoke	73	(41.71)	65	(43.62)	8	(30.77)	0.2826
CDR-SB±SD^**^		1.03	±2.31	0.79	±2.15	2.40	±2.77	0.0009
APOE-ε4+, n (%)		55	(31.43)	49	(32.89)	6	(23.08)	0.3685
WMH, mean IHS±SD	Frontal	0.20	0.47	0.15	±0.25	0.49	±1.03	0.1065
	Temporal*	0.08	0.29	0.04	±0.12	0.32	±0.65	0.0370
	Parietal*	0.16	0.53	0.08	±0.22	0.60	±1.19	0.0357
	Occipital*	0.23	0.42	0.18	±0.29	0.54	±0.78	0.0261
	Total*	0.59	0.79	0.49	±0.57	1.13	±1.44	0.0338
CSF Aβ_1–42_^a^^,^^**^		328.94	153.50	349.5	±148.31	196.74	±118.68	<.0001
CSF ptau181^b^		57.55	38.94	55.2	±38.78	71.9	±37.73	0.0752
CSF Aβ_1–42_:tau ratio ^a^^,^^**^		5.42	4.98	5.91	±5.09	2.32	±2.61	<.0001

^a^Available for n = 141.

^b^Available for n = 144.

*p<0.05;

**p<0.001

We also tested whether the proportion of individuals with microbleeds varied as a function of EYO (i.e., an inflection point). Based on Bayesian information criteria, we did not find evidence of an inflection point in microbleed frequency; the difference in frequency of microbleed status between mutation carriers and non-carriers as a function of EYO was not significant (p = 0.36), although mutation carriers more likely have microbleeds than non-carriers (p = 0.02). The distribution of the microbleed status by EYO group is found in **S1 Table.**

### White matter hyperintensity volume

In this sub-sample of individuals with available microbleed data, we found a similar pattern of differences in WMH volume between mutation carriers and non-carriers as we reported previously[2]: mutation carriers had elevated total and regional WMH, which appeared to diverge approximately 20 years prior to EYO. This pattern remained even after controlling for microbleed status. Presence of microbleeds was associated with increased regional and total WMH volume (Table 2).

**Table 2:** Mixed effect regression coefficients for total and regional WMH volumes and CSF biomarkers.

Outcome	Effect	Estimate	S.E.	DF	t Value	p-value
Frontal lobe WMH volume	Intercept	-0.082	0.24	79	-0.34	0.7342
	Mutation, Carriers	0.153	0.083	89	1.84	0.0688
	Microbleed present^**^	**0.299**	**0.099**	**89**	**3.01**	**0.0034**
	EYO	-0.007	0.007	89	-0.99	0.3255
	EYO* Mutation	0.009	0.007	89	1.24	0.2181
	AGE	0.003	0.005	89	0.56	0.58
	APOE-ε4, Positive	0.064	0.074	89	0.86	0.3925
Temporal lobe WMH volume	Intercept	-0.177	0.148	79	-1.2	0.2354
	Mutation, Carriers	0.097	0.049	89	1.98	0.0507
	Microbleed present^***^	**0.241**	**0.059**	**89**	**4.09**	**<.0001**
	EYO	-0.004	0.004	89	-0.89	0.3733
	EYO* Mutation	0.006	0.004	89	1.28	0.2044
	AGE	0.004	0.003	89	1.17	0.2434
	APOE-ε4, Positive	-0.021	0.044	89	-0.47	0.6372
Parietal lobe WMH volume	Intercept	-0.243	0.268	79	-0.91	0.3669
	Mutation, Carriers^*^	0.197	0.09	89	2.18	0.0319
	Microbleed present^***^	**0.448**	**0.108**	**89**	**4.14**	**<.0001**
	EYO	-0.004	0.007	89	-0.5	0.62
	EYO* Mutation	0.009	0.008	89	1.08	0.281
	AGE	0.005	0.006	89	0.84	0.4051
	APOE-ε4, Positive	0.012	0.081	89	0.15	0.8792
Occipital lobe WMH volume	Intercept	-0.119	0.22	79	-0.54	0.5884
	Mutation, Carriers^**^	**0.223**	**0.072**	**89**	**3.08**	**0.0027**
	Microbleed present^**^	**0.28**	**0.087**	**89**	**3.23**	**0.0017**
	EYO	-0.004	0.006	89	-0.68	0.4992
	EYO* Mutation	0.011	0.006	89	1.68	0.0974
	AGE	0.004	0.005	89	0.81	0.4186
	APOE-ε4, Positive	-0.001	0.066	89	-0.01	0.9904
Total WMH volume	Intercept	0.127	0.418	79	0.3	0.762
	Mutation, Carriers^**^	**0.421**	**0.136**	**89**	**3.08**	**0.0027**
	Microbleed present^**^	**0.507**	**0.164**	**89**	**3.09**	**0.0027**
	EYO	-0.012	0.012	89	-1.08	0.2833
	EYO* Mutation ^*^	**0.028**	**0.012**	**89**	**2.3**	**0.0236**
	AGE	0.002	0.009	89	0.27	0.785
	APOE-ε4, Positive	0.02	0.124	89	0.16	0.8707

*p<0.05;

** p<0.01;

***p<0.001

### Mediation analysis

**Fig 3:**
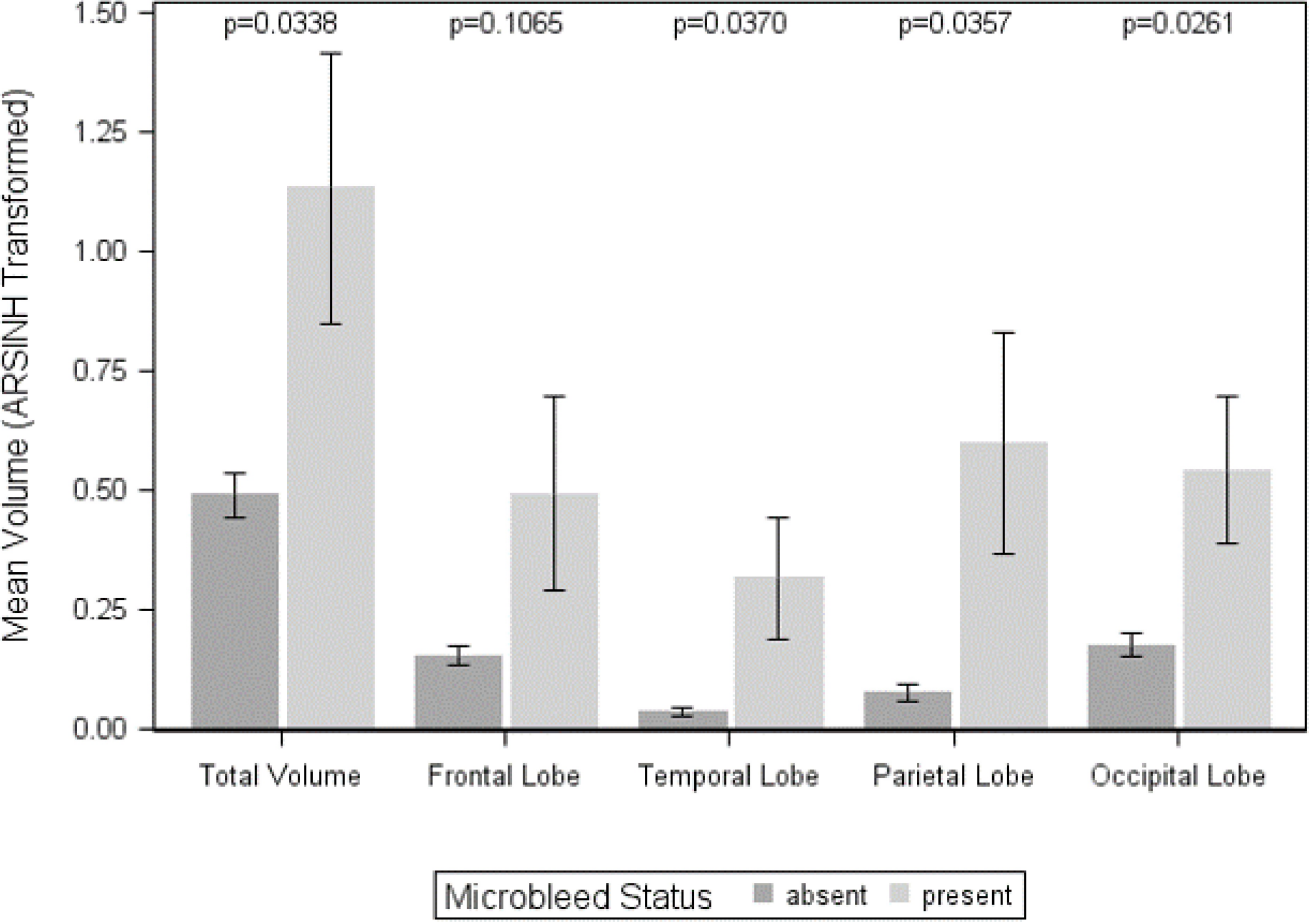
Total and regional WMH volume by microbleed status. Total and regional WMH volumes are expressed in inverse hyperbolic sine transformation units. Individuals with microbleeds had significantly greater total WMH volumes and WMH volumes in temporal, parietal, and occipital lobes.

Individuals with microbleeds had higher total and regional, including temporal, parietal, and occipital WMH volumes than those without microbleeds (Table 1, Fig 3). The 95% confidence intervals of total, direct, and indirect effects were estimated from 1,000 bootstrapped samples. We found that the higher total WMH volume among mutation carriers (total effect, p = 0.0009) was not significantly mediated by microbleed status (indirect effect, p = 0.1389) although it explained approximately 20% of the total effect. Similar patterns were found for the regional WMH volumes (see Table 3). The effect of mutation was moderated by EYO (total effect of EYO*Mutation: p’s<0.05) except for frontal WMH, which had trend level significance (p = 0.05), but there was no significant moderated mediation (indirect effect of EYO*Mutation, p’s>0.29) (Table 4). These findings persist even after controlling for CDR-SB (**S2 Table**). Similar results were found in the subset of CDR-SB is less and equal than 0.5 (**S3 Table**) and after excluding APP participants (**S4 Table**).

**Table 3:** Mediation analysis results without the estimate years of onset adjusted.

	Effect		Estimate	S.E.	95% CI		z	p-value
Total WMHI Volume	total	***	0.3896	0.1172	0.1634	0.6304	3.3248	0.0009
	direct	**	0.3126	0.1116	0.0894	0.5256	2.8011	0.0051
	indirect		0.077	0.0521	-0.0057	0.1974	1.4797	0.1389
Frontal Lobe	total	*	0.1069	0.0484	0.0113	0.2092	2.2075	0.0273
	direct		0.0774	0.043	-0.0048	0.1654	1.7998	0.0719
	indirect		0.0295	0.0243	-0.0056	0.0842	1.214	0.2247
Temporal Lobe	total	**	0.1067	0.0405	0.038	0.1963	2.6351	0.0084
	direct	*	0.0746	0.0321	0.0199	0.1487	2.3238	0.0201
	indirect		0.032	0.0238	-0.0032	0.0867	1.3486	0.1775
Parietal Lobe	total	**	0.2207	0.074	0.0937	0.3846	2.9843	0.0028
	direct	**	0.158	0.0562	0.064	0.2833	2.8118	0.0049
	indirect		0.0628	0.044	-0.0045	0.163	1.4259	0.1539
Occipital Lobe	total	***	0.2378	0.0643	0.12	0.3801	3.6973	0.0002
	direct	**	0.1965	0.0606	0.0871	0.3248	3.2438	0.0012
	indirect		0.0413	0.0265	-0.0013	0.1051	1.5599	0.1188

*p<0.05;

**p<0.01;

***p<0.001;

All models were adjusted for APOE-4 and age.

**Table 4:** Covariate adjusted total, indirect and direct effects of mutation status. APOE-4 and age were adjusted in all the models. The 95% confidence intervals of total, direct and indirect effect were estimated from 1000 bootstrapped samples.

	Effect			Estimate	S.E.	95% CI		z	p-value
Total WMH volume	total	EYO*Mutation	**	0.0313	0.0105	0.0113	0.0526	2.9924	0.0028
		Mutation	***	0.5541	0.1438	0.2862	0.8461	3.8529	0.0001
	direct	EYO* Mutation	**	0.028	0.0107	0.0073	0.0492	2.6129	0.009
		Mutation	***	0.4672	0.1392	0.1883	0.7449	3.356	0.0008
	indirect	EYO* Mutation		0.0033	0.0036	-0.0024	0.0117	0.9217	0.3567
		Mutation		0.0869	0.0615	-0.013	0.2269	1.414	0.1574
Frontal Lobe	total	EYO* Mutation		0.0105	0.0054	-0.0003	0.0211	1.9463	0.0516
		Mutation	**	0.1615	0.0625	0.0321	0.289	2.5852	0.0097
	direct	EYO* Mutation		0.0093	0.0052	-0.0013	0.0196	1.7901	0.0734
		Mutation	*	0.1271	0.0551	0.0161	0.2345	2.3081	0.021
	indirect	EYO* Mutation		0.0012	0.0018	-0.0016	0.006	0.6843	0.4938
		Mutation		0.0344	0.0285	-0.0082	0.1009	1.2081	0.227
Temporal Lobe	total	EYO* Mutation	*	0.0089	0.0036	0.0024	0.0172	2.4608	0.0139
		Mutation	**	0.1546	0.0532	0.0667	0.2708	2.9074	0.0036
	direct	EYO* Mutation		0.0076	0.0037	0.001	0.0159	2.0257	0.0428
		Mutation	**	0.1167	0.044	0.0447	0.2141	2.6532	0.008
	indirect	EYO* Mutation		0.0014	0.0019	-0.0016	0.0055	0.74	0.4593
		Mutation		0.0379	0.0288	-0.0041	0.1036	1.3172	0.1878
Parietal Lobe	total	EYO* Mutation	*	0.0132	0.0061	0.0022	0.0261	2.1683	0.0301
		Mutation	**	0.291	0.0957	0.1232	0.4993	3.0405	0.0024
	direct	EYO* Mutation		0.0097	0.006	-0.0014	0.023	1.6174	0.1058
		Mutation	**	0.2121	0.0735	0.0913	0.3714	2.8841	0.0039
	indirect	EYO* Mutation		0.0035	0.0033	-0.0022	0.0114	1.0586	0.2898
		Mutation		0.0789	0.0536	-0.0045	0.2017	1.4711	0.1413
Occipital Lobe	total	EYO* Mutation	*	0.0132	0.0055	0.0027	0.0239	2.4168	0.0157
		Mutation	***	0.308	0.0757	0.1683	0.4728	4.0668	<0.0001
	direct	EYO* Mutation	*	0.0113	0.0057	0.0005	0.0221	1.987	0.0469
		Mutation	***	0.2602	0.0728	0.1246	0.4212	3.5762	0.0003
	indirect	EYO* Mutation		0.0019	0.0018	-0.0008	0.0062	1.0218	0.3069
		Mutation		0.0477	0.0316	-0.0045	0.1216	1.5093	0.1312

*p<0.05;

**p<0.01;

***p<0.001;

All models were adjusted for APOE-4 and age.

Finally, when we limited analyses to those individuals with microbleeds (n = 26) and tested whether the number of microbleeds mediated the relationship between mutation status and regional or global WMH volume, we did not find evidence of a mediational relationship (p’s > 0.63).

### Discussion

Several important observations emerged from this work among individuals with and without genetic mutations for AD. First, the primary aim of this study was to determine the extent to which the increased WMH among mutation carriers reflects, or is mediated by, increases in vascular forms of beta amyloid, which we operationally defined as presence of cerebral microbleeds. Although we observed some co-dependency between WMH and cerebral microbleeds, the increased WMH volume among mutation carriers was only modestly mediated by this marker of cerebral amyloid angiopathy. Second, those with microbleeds had more severe AD fluid biomarker profiles, defined as lower levels of CSF Aβ_42_ and lower CSF Aβ_42_/tau ratios. Third, individuals with mutations were more likely to have cerebral microbleeds than those without mutations, although this increased likelihood did not vary as a function of estimated time to symptom onset, perhaps due to a relatively small sample size **(S4 Table).** Finally, we were able to reproduce our previous finding[2] of increased WMH volume among mutation carriers up to 20 years prior to expected symptom onset in this subset of participants with available GRE scans.

There is considerable debate about the role of cerebrovascular disease in AD onset and pathogenesis. Current pathogenic models of the disease[16] and their application to clinical diagnostic criteria[17–19] emphasize a single pathway that begins with fibrillar forms of (parenchymal) beta amyloid, followed by the deposition of tau pathology, which subsequently results in neurodegeneration and clinical symptoms. But there is ample evidence of involvement of cerebrovascular disease in AD. Postmortem studies consistently demonstrate that the presence of cerebrovascular lesions among symptomatic individuals with AD is much more common than not and suggest that pathologies, including cerebrovascular disease, in addition to primary AD pathology, lower the threshold, increase risk, or are perhaps necessary for the disease to express clinically[20]. *In vivo* neuroimaging studies support a similar conclusion: up to one third of older adults with no apparent symptomatology have evidence of amyloid deposition sufficient enough to meet pathological criteria for AD[21], but increased markers of cerebrovascular disease seem to distinguish symptomatic individuals with amyloidosis from asymptomatic individuals with amyloidosis[22]. A common interpretation of such findings is that cerebrovascular disease represents a comorbidity that lowers the clinical diagnostic threshold for AD and contributes additively to disease presentation. The opportunity to test the extent to which cerebrovascular disease is playing a role among individuals with autosomal dominant genetic mutations for AD offers the unique opportunity to test the extent to which cerebrovascular disease is a “core” feature of AD; the work here, combined with our previous observation[2], provides evidence that WMH are not merely a comorbidity or confounding factor but rather reflect primary pathological features of the disease, present in individuals genetically-determined to develop the disease. Participants in this study were all of equal likelihood of inheriting an autosomal dominant mutation for AD and were essentially “randomly assigned” to the carrier or non-carrier groups, thus providing experimental evidence for increased WMH in genetically-determined AD.

White matter hyperintensities are typically considered markers of small vessel cerebrovascular disease and postmortem correlates studies suggest several changes secondary to ischemic injury, including demyelination, axonal loss, blood barrier dysfunction, and arteriolosclerosis[3]. Because of the high levels of amyloidosis among individuals with autosomal mutations for AD[1], the extent to which WMH reflects CAA is a possibility that motivated the current study. There is understandably little information about pathological changes in the white matter of individuals with autosomal dominant forms of AD, considering the low prevalence of individuals with autosomal forms of AD. In one study, WMH volume determined *in vivo* did correlate modestly with CAA restricted to the temporal lobes, but also correlated with leptomeningeal blood vessel diameter and lower density of CD68-positive microglia in posterior brain regions, where the impact of WMH is greatest[2, 4]. The findings here also suggest that the increased observed WMH in autosomal dominant AD is not attributable entirely to CAA. It is important to note that among autosomal dominant mutation carriers for AD, the pathophysiological basis of WMH has not been elucidated clearly, and could include damage due to ischemia, as is observed in aging, or to other factors such as blood brain barrier dysfunction, Wallerian-like degeneration secondary to neurodegeneration, and inflammation. Thus, we speculate that the role of WMH in autosomal dominant AD reflects either the primary involvement of vascular damage in AD or the manifestation of other pernicious factors, such as inflammation, in AD. Indeed, a recent study showed some preliminary evidence of increased T-lymphocytic responses in the postmortem white matter tissue of PSEN1 mutation carriers[23]. Nonetheless, our results suggest that both radiological phenomena—- microbleeds and WMH—- are important features of AD. Future work should therefore consider more explicitly the underlying mechanisms as potential drivers of disease pathogenesis and treatment targets as opposed to comorbidities not central to the disease.

Cerebral microbleeds in the current study were measured on GRE 3T MRI scans. These scans are known to have good specificity for microbleed detection but relatively lower sensitivity compared with susceptibility weighted images and higher field strength magnets. Relatively few microbleeds were detected and it is possible that some participants had microbleeds that were undetected by the technology employed, thus somewhat biasing our analyses towards the null hypothesis and producing Type II statistical errors. On the other hand, the high specificity of microbleed detection on GRE gives us confidence that individuals identified as having microbleeds were accurately classified and have evidence of the presence of CAA. When we restricted the sample to only those participants with detectable microbleeds, we also did not find a mediating role of CAA in the relationship between mutation status and WMH volume, which supports our overall observation, although relatively low sample size could also bias the findings somewhat towards the null. Furthermore, microbleeds that distributed in subcortical/deep regions are typically attributed to hypertensive pathology whereas microbleeds distributed in lobar regions are typically attributed to CAA. Not surprising for the age and population under study, microbleeds in the current sample tended to be in lobar regions. Only two participants had exclusively deep microbleeds. We re-ran the analyses coding these individuals has having microbleed status of “absent” and the results remained essentially unchanged (data not shown).

The development of disease modifying interventions for AD has resulted in a number of disappointing results[24]. Recent trials that focused on treatments that attempt to clear fibrillar amyloid pathology or prevent its deposition yielded negative findings with respect to clinical outcomes and removal of amyloid, despite some moderate evidence of target engagement[24]. There has been little focus on vascular mechanisms as feasible targets for treatment of prevention strategies. This work adds to a growing body of literature that clearly implicates WMH in AD; future work should consider mechanisms of WMH including cerebrovascular disease and interventions that focus on cerebrovascular disease in AD.

## Supporting information

S1 Table Distribution of microbleed status by age group. There was a trend of increasing microbleeds as the EYO increases (chi-square test p-value: 0.08). However, after adjusting for family, age at visit, and E4, there was no significant increasing trend.
(DOCX)

S2 Table Mediation and moderated mediation results with CDR-SB as a covariate (n = 175). In addition to CDR-SB, age, EYO and ApoE-4 was controlled.
(DOCX)

S3 Table Mediation and moderated mediation results for the subset of CDR-SB≤.5 (n = 134). In the models, age, EYO and ApoE-4 was controlled.
(DOCX)

S4 Table Mediation and moderated mediation results without APP subtype (n = 138). In the models, age, EYO and ApoE-4 was controlled.
(DOCX)
