# Supplementary material for: White matter hyperintensities and the mediating role of cerebral amyloid angiopathy in dominantly-inherited Alzheimer’s disease
Source: PLoS One. 2018 May 9;13(5):e0195838. doi: 10.1371/journal.pone.0195838 (PMC5942789; doi:10.1371/journal.pone.0195838)
Supplement: S1 Table — There was a trend of increasing microbleeds as the EYO increases (chi-square test p-value: 0.08). However, after adjusting for family, age at visit, and E4, there was no significant increasing trend. (DOCX) [file pone.0195838.s001.docx]

**S1 Table. Distribution of microbleed status by age group**. There was a trend of increasing microbleeds as the EYO increases (chi-square test p-value: 0.08). However, after adjusting for family, age at visit, and E4, there was no significant increasing trend.

| EYO | Microbleed Status | | | |
| --- | --- | --- | --- | --- |
|  | Absent (n=149) | | Present (n=26) | |
|  | N | % | N | % |
| [-30,-20) | 9 | 100 | 0 | 0 |
| [-20,-10) | 38 | 88.37 | 5 | 11.63 |
| [-10,0) | 66 | 89.19 | 8 | 10.81 |
| [0,10) | 29 | 74.36 | 10 | 25.64 |
| [10,21]) | 7 | 70 | 3 | 30 |
