# Supplementary material for: White matter hyperintensities and the mediating role of cerebral amyloid angiopathy in dominantly-inherited Alzheimer’s disease
Source: PLoS One. 2018 May 9;13(5):e0195838. doi: 10.1371/journal.pone.0195838 (PMC5942789; doi:10.1371/journal.pone.0195838)
Supplement: S2 Table — In addition to CDR-SB, age, EYO and ApoE-4 was controlled. (DOCX) [file pone.0195838.s002.docx]

**S2 Table. Mediation and moderated mediation results with CDR-SB as a covariate (n=175)**. In addition to CDR-SB, age, EYO and ApoE-4 was controlled.

| ROI | Effects | | Estimate | 95% CI | | z | p |
| --- | --- | --- | --- | --- | --- | --- | --- |
| Total WMH Volume | total | ** | 0.3542 | 0.1136 | 0.6052 | 2.8484 | 0.0044 |
|  | direct | * | 0.2899 | 0.0632 | 0.5151 | 2.5770 | 0.0100 |
|  | indirect |  | 0.0643 | -0.0079 | 0.1872 | 1.2842 | 0.1991 |
| Frontal Lobe | total |  | 0.0948 | 0.0006 | 0.2043 | 1.7981 | 0.0722 |
|  | direct |  | 0.0691 | -0.0170 | 0.1636 | 1.5154 | 0.1297 |
|  | indirect |  | 0.0257 | -0.0081 | 0.0800 | 1.0862 | 0.2774 |
| Temporal Lobe | total | * | 0.1074 | 0.0282 | 0.2045 | 2.3559 | 0.0185 |
|  | direct | * | 0.0798 | 0.0207 | 0.1605 | 2.2481 | 0.0246 |
|  | indirect |  | 0.0276 | -0.0047 | 0.0842 | 1.1989 | 0.2306 |
| Parietal Lobe | total | ** | 0.2164 | 0.0712 | 0.3952 | 2.5989 | 0.0094 |
|  | direct | ** | 0.1649 | 0.0557 | 0.3033 | 2.6155 | 0.0089 |
|  | indirect |  | 0.0515 | -0.0086 | 0.1556 | 1.1932 | 0.2328 |
| Occipital Lobe | total | *** | 0.2246 | 0.1018 | 0.3750 | 3.3491 | 0.0008 |
|  | direct | ** | 0.1900 | 0.0769 | 0.3203 | 3.1006 | 0.0019 |
|  | indirect |  | 0.0346 | -0.0033 | 0.0995 | 1.3519 | 0.1764 |

| ROI | Effects | |  | Estimate | 95% CI | | z | p |
| --- | --- | --- | --- | --- | --- | --- | --- | --- |
| Total WMH volume | total | EYO*MUTATION | * | 0.0307 | 0.0080 | 0.0550 | 2.5759 | 0.0100 |
|  |  | MUTATION | ** | 0.5443 | 0.2436 | 0.9103 | 3.2502 | 0.0012 |
|  | direct | EYO*MUTATION | * | 0.0284 | 0.0053 | 0.0525 | 2.3507 | 0.0187 |
|  |  | MUTATION | ** | 0.4699 | 0.1821 | 0.7667 | 3.0387 | 0.0024 |
|  | indirect | EYO*MUTATION |  | 0.0023 | -0.0044 | 0.0111 | 0.6221 | 0.5339 |
|  |  | MUTATION |  | 0.0743 | -0.0114 | 0.2176 | 1.2400 | 0.2150 |
| Frontal Lobe | total | EYO*MUTATION |  | 0.0103 | -0.0014 | 0.0229 | 1.6732 | 0.0943 |
|  |  | MUTATION | * | 0.1574 | 0.0225 | 0.3180 | 2.1111 | 0.0348 |
|  | direct | EYO*MUTATION |  | 0.0094 | -0.0021 | 0.0222 | 1.5672 | 0.1171 |
|  |  | MUTATION |  | 0.1270 | 0.0018 | 0.2588 | 1.9350 | 0.0530 |
|  | indirect | EYO*MUTATION |  | 0.0009 | -0.0023 | 0.0057 | 0.4841 | 0.6283 |
|  |  | MUTATION |  | 0.0304 | -0.0104 | 0.0942 | 1.0733 | 0.2832 |
| Temporal Lobe | total | EYO*MUTATION | * | 0.0099 | 0.0021 | 0.0199 | 2.1903 | 0.0285 |
|  |  | MUTATION | * | 0.1691 | 0.0528 | 0.3180 | 2.5314 | 0.0114 |
|  | direct | EYO*MUTATION |  | 0.0090 | 0.0012 | 0.0190 | 1.9431 | 0.0520 |
|  |  | MUTATION | * | 0.1363 | 0.0431 | 0.2585 | 2.4572 | 0.0140 |
|  | indirect | EYO*MUTATION |  | 0.0009 | -0.0032 | 0.0054 | 0.4508 | 0.6521 |
|  |  | MUTATION |  | 0.0327 | -0.0056 | 0.1004 | 1.1627 | 0.2449 |
| Parietal Lobe | total | EYO*MUTATION |  | 0.0141 | 0.0011 | 0.0309 | 1.8491 | 0.0644 |
|  |  | MUTATION | * | 0.3043 | 0.0966 | 0.5717 | 2.5514 | 0.0107 |
|  | direct | EYO*MUTATION |  | 0.0116 | -0.0011 | 0.0286 | 1.5806 | 0.1140 |
|  |  | MUTATION | * | 0.2386 | 0.0817 | 0.4467 | 2.5537 | 0.0107 |
|  | indirect | EYO*MUTATION |  | 0.0025 | -0.0044 | 0.0106 | 0.7229 | 0.4697 |
|  |  | MUTATION |  | 0.0656 | -0.0068 | 0.1931 | 1.2300 | 0.2187 |
| Occipital Lobe | total | EYO*MUTATION | * | 0.0131 | 0.0019 | 0.0257 | 2.1722 | 0.0298 |
|  |  | MUTATION | *** | 0.3061 | 0.1550 | 0.4915 | 3.6299 | 0.0003 |
|  | direct | EYO*MUTATION |  | 0.0118 | -0.0003 | 0.0245 | 1.9002 | 0.0574 |
|  |  | MUTATION | *** | 0.2653 | 0.1232 | 0.4382 | 3.3920 | 0.0007 |
|  | indirect | EYO*MUTATION |  | 0.0014 | -0.0020 | 0.0059 | 0.7139 | 0.4753 |
|  |  | MUTATION |  | 0.0408 | -0.0042 | 0.1133 | 1.3200 | 0.1868 |
