# Supplementary material for: White matter hyperintensities and the mediating role of cerebral amyloid angiopathy in dominantly-inherited Alzheimer’s disease
Source: PLoS One. 2018 May 9;13(5):e0195838. doi: 10.1371/journal.pone.0195838 (PMC5942789; doi:10.1371/journal.pone.0195838)
Supplement: S3 Table — In the models, age, EYO and ApoE-4 was controlled. (DOCX) [file pone.0195838.s003.docx]

**S3 Table. Mediation and moderated mediation results for the subset of CDR-SB≤.5 (n=134**). In the models, age, EYO and ApoE-4 was controlled.

| ROI | Effects |  | Estimate | 95% CI | | z | p |
| --- | --- | --- | --- | --- | --- | --- | --- |
| Total WMH Volume | total | * | 0.2670 | 0.0341 | 0.5248 | 2.2154 | 0.0267 |
|  | direct |  | 0.2064 | 0.0091 | 0.4214 | 1.9497 | 0.0512 |
|  | indirect |  | 0.0606 | -0.0191 | 0.2049 | 1.0328 | 0.3017 |
| Frontal Lobe | total |  | 0.0790 | -0.0086 | 0.1786 | 1.6582 | 0.0973 |
|  | direct |  | 0.0638 | -0.0137 | 0.1526 | 1.4849 | 0.1376 |
|  | indirect |  | 0.0152 | -0.0093 | 0.0633 | 0.8170 | 0.4139 |
| Temporal Lobe | total |  | 0.0627 | 0.0090 | 0.1576 | 1.6182 | 0.1056 |
|  | direct |  | 0.0460 | 0.0061 | 0.1143 | 1.6699 | 0.0949 |
|  | indirect |  | 0.0167 | -0.0081 | 0.0703 | 0.7870 | 0.4313 |
| Parietal Lobe | total |  | 0.1465 | 0.0199 | 0.3257 | 1.8258 | 0.0679 |
|  | direct | * | 0.1096 | 0.0191 | 0.2331 | 1.9779 | 0.0479 |
|  | indirect |  | 0.0369 | -0.0129 | 0.1452 | 0.8548 | 0.3927 |
| Occipital Lobe | total | * | 0.1630 | 0.0411 | 0.3000 | 2.4694 | 0.0135 |
|  | direct | * | 0.1346 | 0.0273 | 0.2551 | 2.3190 | 0.0204 |
|  | indirect |  | 0.0283 | -0.0072 | 0.0971 | 0.9973 | 0.3186 |

| ROI | Effects | | | Estimate | 95% CI | | z | p |
| --- | --- | --- | --- | --- | --- | --- | --- | --- |
| Total WMH volume | total | EYO*MUTATION | * | 0.0308 | 0.0073 | 0.0571 | 2.5237 | 0.0116 |
|  |  | MUTATION | ** | 0.4841 | 0.1494 | 0.8416 | 2.7343 | 0.0063 |
|  | direct | EYO*MUTATION | * | 0.0282 | 0.0052 | 0.0560 | 2.3053 | 0.0211 |
|  |  | MUTATION | ** | 0.4088 | 0.1295 | 0.7417 | 2.6121 | 0.0090 |
|  | indirect | EYO*MUTATION |  | 0.0026 | -0.0054 | 0.0150 | 0.5255 | 0.5993 |
|  |  | MUTATION |  | 0.0753 | -0.0241 | 0.2655 | 0.9957 | 0.3194 |
| Frontal Lobe | total | EYO*MUTATION |  | 0.0119 | -0.0001 | 0.0244 | 1.9416 | 0.0522 |
|  |  | MUTATION | * | 0.1621 | 0.0246 | 0.3234 | 2.0888 | 0.0367 |
|  | direct | EYO*MUTATION |  | 0.0113 | -0.0006 | 0.0238 | 1.8719 | 0.0612 |
|  |  | MUTATION | * | 0.1445 | 0.0161 | 0.2917 | 1.9995 | 0.0456 |
|  | indirect | EYO*MUTATION |  | 0.0006 | -0.0019 | 0.0043 | 0.3849 | 0.7003 |
|  |  | MUTATION |  | 0.0176 | -0.0144 | 0.0768 | 0.7437 | 0.4571 |
| Temporal Lobe | total | EYO*MUTATION |  | 0.0068 | 0.0000 | 0.0166 | 1.4658 | 0.1427 |
|  |  | MUTATION |  | 0.1116 | 0.0097 | 0.2577 | 1.6539 | 0.0981 |
|  | direct | EYO*MUTATION |  | 0.0059 | -0.0001 | 0.0153 | 1.3688 | 0.1711 |
|  |  | MUTATION |  | 0.0890 | 0.0099 | 0.2095 | 1.7272 | 0.0841 |
|  | indirect | EYO*MUTATION |  | 0.0009 | -0.0017 | 0.0058 | 0.5139 | 0.6073 |
|  |  | MUTATION |  | 0.0226 | -0.0104 | 0.0975 | 0.7986 | 0.4245 |
| Parietal Lobe | total | EYO*MUTATION |  | 0.0113 | 0.0004 | 0.0274 | 1.5188 | 0.1288 |
|  |  | MUTATION |  | 0.2281 | 0.0297 | 0.4937 | 1.7872 | 0.0739 |
|  | direct | EYO*MUTATION |  | 0.0092 | -0.0002 | 0.0250 | 1.3435 | 0.1791 |
|  |  | MUTATION |  | 0.1774 | 0.0266 | 0.3925 | 1.8662 | 0.0620 |
|  | indirect | EYO*MUTATION |  | 0.0021 | -0.0033 | 0.0122 | 0.5594 | 0.5759 |
|  |  | MUTATION |  | 0.0507 | -0.0150 | 0.2032 | 0.8647 | 0.3872 |
| Occipital Lobe | total | EYO*MUTATION |  | 0.0108 | 0.0005 | 0.0224 | 1.8790 | 0.0602 |
|  |  | MUTATION | ** | 0.2408 | 0.0709 | 0.4227 | 2.6715 | 0.0076 |
|  | direct | EYO*MUTATION |  | 0.0095 | -0.0007 | 0.0216 | 1.6260 | 0.1040 |
|  |  | MUTATION | ** | 0.2041 | 0.0596 | 0.3683 | 2.5922 | 0.0095 |
|  | indirect | EYO*MUTATION |  | 0.0013 | -0.0021 | 0.0070 | 0.5691 | 0.5693 |
|  |  | MUTATION |  | 0.0367 | -0.0085 | 0.1327 | 0.9771 | 0.3285 |
