# Supplementary material for: White matter hyperintensities and the mediating role of cerebral amyloid angiopathy in dominantly-inherited Alzheimer’s disease
Source: PLoS One. 2018 May 9;13(5):e0195838. doi: 10.1371/journal.pone.0195838 (PMC5942789; doi:10.1371/journal.pone.0195838)
Supplement: S4 Table — In the models, age, EYO and ApoE-4 was controlled. (DOCX) [file pone.0195838.s004.docx]

**S4 Table. Mediation and moderated mediation results without APP subtype (n=138)**. In the models, age, EYO and ApoE-4 was controlled.

| ROI | Effects |  | Estimate | 95% CI | | z | p |
| --- | --- | --- | --- | --- | --- | --- | --- |
| Total WMH volume | total |  | 0.2145 | -0.042 | 0.4663 | 1.7297 | 0.0837 |
|  | direct |  | 0.2008 | -0.057 | 0.4776 | 1.4701 | 0.1415 |
|  | indirect |  | 0.0138 | -0.063 | 0.0866 | 0.3815 | 0.7029 |
| Frontal Lobe | total |  | 0.0506 | -0.048 | 0.1542 | 0.9822 | 0.3260 |
|  | direct |  | 0.0448 | -0.057 | 0.1524 | 0.8361 | 0.4031 |
|  | indirect |  | 0.0059 | -0.039 | 0.0572 | 0.2418 | 0.8090 |
| Temporal Lobe | total | * | 0.0639 | 0.0097 | 0.1333 | 2.0008 | 0.0454 |
|  | direct |  | 0.0613 | 0.0058 | 0.1396 | 1.7788 | 0.0753 |
|  | indirect |  | 0.0025 | -0.017 | 0.0222 | 0.2639 | 0.7919 |
| Parietal Lobe | total | * | 0.1328 | 0.0318 | 0.2559 | 2.3231 | 0.0202 |
|  | direct |  | 0.1133 | 0.0105 | 0.2383 | 1.9482 | 0.0514 |
|  | indirect |  | 0.0195 | -0.021 | 0.0814 | 0.7395 | 0.4596 |
| Occipital Lobe | total | * | 0.1534 | 0.029 | 0.2894 | 2.3090 | 0.0209 |
|  | direct | * | 0.1381 | 0.0077 | 0.2814 | 1.9736 | 0.0484 |
|  | indirect |  | 0.0153 | -0.03 | 0.065 | 0.6461 | 0.5182 |

| ROI | Effects | Effect |  | mean | 95% CI | | z | p |
| --- | --- | --- | --- | --- | --- | --- | --- | --- |
| Total WMH volume | total | EYO*MUTATION | ** | 0.0328 | 0.0094 | 0.0595 | 2.6369 | 0.0084 |
|  |  | MUTATION | ** | 0.5752 | 0.2513 | 0.9605 | 3.2338 | 0.0012 |
|  | direct | EYO*MUTATION | * | 0.0298 | 0.0063 | 0.0550 | 2.3600 | 0.0183 |
|  |  | MUTATION | ** | 0.4895 | 0.1872 | 0.8081 | 2.9772 | 0.0029 |
|  | indirect | EYO*MUTATION |  | 0.0031 | -0.0034 | 0.0131 | 0.7842 | 0.4329 |
|  |  | MUTATION |  | 0.0857 | -0.0120 | 0.2338 | 1.3252 | 0.1851 |
| Frontal Lobe | total | EYO*MUTATION |  | 0.0110 | -0.0005 | 0.0239 | 1.7778 | 0.0754 |
|  |  | MUTATION | * | 0.1668 | 0.0240 | 0.3340 | 2.1674 | 0.0302 |
|  | direct | EYO*MUTATION |  | 0.0097 | -0.0018 | 0.0230 | 1.6214 | 0.1049 |
|  |  | MUTATION |  | 0.1314 | 0.0001 | 0.2650 | 1.9427 | 0.0521 |
|  | indirect | EYO*MUTATION |  | 0.0013 | -0.0020 | 0.0064 | 0.6479 | 0.5170 |
|  |  | MUTATION |  | 0.0354 | -0.0101 | 0.1053 | 1.1416 | 0.2536 |
| Temporal Lobe | total | EYO*MUTATION | * | 0.0107 | 0.0025 | 0.0206 | 2.2757 | 0.0229 |
|  |  | MUTATION | ** | 0.1804 | 0.0608 | 0.3372 | 2.5895 | 0.0096 |
|  | direct | EYO*MUTATION | * | 0.0095 | 0.0015 | 0.0202 | 1.9632 | 0.0496 |
|  |  | MUTATION |  | 0.1434 | 0.0506 | 0.2718 | 2.4898 | 0.0128 |
|  | indirect | EYO*MUTATION |  | 0.0012 | -0.0031 | 0.0059 | 0.5619 | 0.5742 |
|  |  | MUTATION |  | 0.0370 | -0.0052 | 0.1080 | 1.2198 | 0.2226 |
| Parietal Lobe | total | EYO*MUTATION | * | 0.0158 | 0.0027 | 0.0332 | 2.0115 | 0.0443 |
|  |  | MUTATION | ** | 0.3304 | 0.1173 | 0.6076 | 2.6613 | 0.0078 |
|  | direct | EYO*MUTATION |  | 0.0127 | 0.0001 | 0.0301 | 1.6811 | 0.0928 |
|  |  | MUTATION | ** | 0.2554 | 0.0922 | 0.4665 | 2.6360 | 0.0084 |
|  | indirect | EYO*MUTATION |  | 0.0031 | -0.0036 | 0.0115 | 0.8583 | 0.3907 |
|  |  | MUTATION |  | 0.0749 | -0.0066 | 0.2070 | 1.3234 | 0.1857 |
| Occipital Lobe | total | EYO*MUTATION | * | 0.0142 | 0.0024 | 0.0277 | 2.2788 | 0.0227 |
|  |  | MUTATION | *** | 0.3226 | 0.1642 | 0.5125 | 3.6648 | 0.0002 |
|  | direct | EYO*MUTATION |  | 0.0125 | -0.0001 | 0.0257 | 1.9355 | 0.0529 |
|  |  | MUTATION | *** | 0.2759 | 0.1275 | 0.4513 | 3.3537 | 0.0008 |
|  | indirect | EYO*MUTATION |  | 0.0018 | -0.0014 | 0.0065 | 0.8799 | 0.3789 |
|  |  | MUTATION |  | 0.0466 | -0.0046 | 0.1249 | 1.4085 | 0.1590 |
